# Surgical Management of a Left Main Coronary Trunk-Adjacent Functional Cardiac Paraganglioma Using Proactive Coronary Artery Bypass Grafting: A Case Report

**DOI:** 10.70352/scrj.cr.26-0056

**Published:** 2026-04-16

**Authors:** Kenichi Morimoto, Yuichiro Kishimoto, Takeshi Onohara, Hiromu Horie, Tsuyoshi Sasami, Naoki Sumi, Nozomi Kishimoto, Junpei Tokutome, Junya Nakashima, Yasushi Yoshikawa

**Affiliations:** Department of Cardiovascular Surgery, Tottori University Faculty of Medicine, Yonago, Tottori, Japan

**Keywords:** cardiac paraganglioma, left main coronary trunk, en bloc resection, coronary artery bypass grafting

## Abstract

**INTRODUCTION:**

Cardiac paragangliomas are exceptionally rare and often functional tumors that frequently arise near the aortic root and proximal coronary arteries. Complete excision is the only potentially curative treatment, but lesions adjacent to the left main coronary trunk (LMT) pose a major surgical dilemma: achieving oncologic radicality while preserving coronary perfusion and controlling massive bleeding.

**CASE PRESENTATION:**

A 33-year-old man presented with episodic postprandial chest/abdominal pain, paroxysmal hypertension, and cold sweating. Imaging revealed a hypervascular cardiac mass located between the ascending aorta and main pulmonary artery, extending to the left atrial roof; coronary angiography demonstrated tumor-feeding branches from the left anterior descending artery (LAD) and right coronary artery. After preoperative α-adrenergic blockade with doxazosin, surgery was performed via median sternotomy with cardiopulmonary bypass (CPB). Given the tumor’s proximity to the LMT and the anticipated risk of compromised coronary perfusion to achieve macroscopic complete resection, planned coronary artery bypass grafting was performed before tumor excision. Under cardioplegic arrest, both the ascending aorta and main pulmonary artery were transected for exposure. The LMT and LAD were preserved, whereas the left circumflex artery coursed through the tumor and was sacrificed. En bloc resection including part of the left atrial roof was required, followed by bovine pericardial patch reconstruction. Diffuse massive bleeding from the left atrial patch suture line and the dissection surface required a second CPB run for hemostasis, and recurrent ventricular tachycardia/fibrillation after weaning from CPB necessitated temporary rescue peripheral veno-arterial extracorporeal membrane oxygenation, which was weaned off on POD3. Postoperative catecholamine levels normalized, and ^123^I-metaiodobenzylguanidine scintigraphy demonstrated no abnormal uptake at the cardiac operative site, consistent with complete resection.

**CONCLUSIONS:**

For functional cardiac paragangliomas adjacent to the LMT, integrating planned revascularization before tumor manipulation can provide a myocardial “safety net” that enables oncologically oriented en bloc resection when coronary sacrifice becomes unavoidable. This operation carries an exceptionally high risk of massive bleeding; therefore, meticulous hemostatic planning and preparedness—including a low threshold for prompt re-institution of CPB—may be crucial for the safe completion of radical resection.

## Abbreviations

CABG
: coronary artery bypass grafting
CPB
: cardiopulmonary bypass
ECMO
: extracorporeal membrane oxygenation
GAPP
: Grading of Adrenal Pheochromocytoma and Paraganglioma
LAD
: left anterior descending artery
LM
: left main coronary artery
LMT
: left main coronary trunk
PL
: posterolateral
RCA
: right coronary artery
SDHB
: succinate dehydrogenase subunit B
SDHx
: succinate dehydrogenase complex
SVG
: saphenous vein graft
VA-ECMO
: veno-arterial extracorporeal membrane oxygenation.

## INTRODUCTION

Cardiac paragangliomas are exceptionally rare neuroendocrine tumors arising from intrapericardial paraganglia. Most are functional and present in young to middle-aged adults with symptoms of catecholamine excess, and many arise near the aortic root and proximal coronary arteries.^1–3)^ Complete surgical excision is the only potentially curative treatment; however, surgery is technically demanding because radical resection must be balanced against catastrophic bleeding and perioperative myocardial ischemia, particularly for tumors adjacent to the LMT.^1–5)^ Importantly, operative mortality remains non-negligible even in contemporary series, and perioperative death due to hemorrhagic complications has been reported, underscoring the potentially lethal nature of LMT-adjacent disease.^1,3,5)^ We report a case of functional cardiac paraganglioma located between the ascending aorta and main pulmonary artery, infiltrating the left atrial roof and encasing the circumflex artery, in which a planned strategy of proactive coronary revascularization before tumor manipulation and en bloc resection enabled complete excision while preserving coronary perfusion.

## CASE PRESENTATION

A 33-year-old man with no significant past medical history was referred for surgical management of a cardiac mass detected during evaluation of episodic postprandial chest and abdominal pain accompanied by paroxysmal hypertension and cold sweating. He had no history of hypertension, diabetes, dyslipidemia, smoking, or alcohol use. His family history was notable for the sudden death of his grandmother and mother at a young age.

**Fig. 1:**
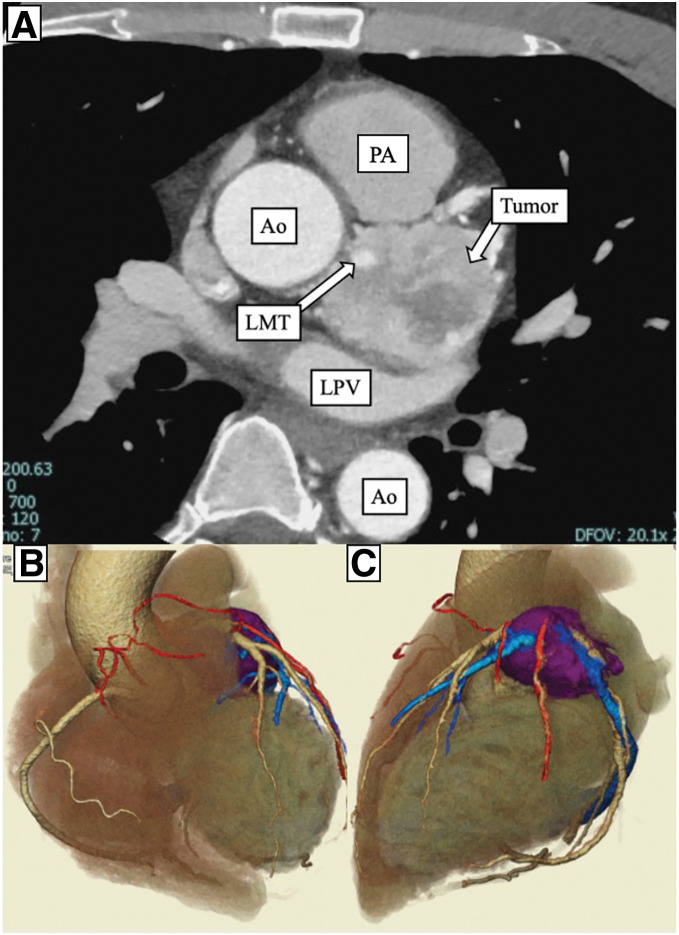
Preoperative contrast-enhanced CT and 3D reconstruction. (**A**) Axial contrast-enhanced CT shows a heterogeneous hypervascular mass adjacent to the aortic root and left atrial roof, in close proximity to the LMT. (**B**) 3D volume-rendered CT (anterior view) depicts the tumor (purple) between the ascending Ao and main PA with tumor-feeding arteries (red) and draining veins (blue). (**C**) Left lateral view further illustrates the tumor–great vessel relationship and vascular connections. Ao, aorta; LMT, left main coronary trunk; LPV, left pulmonary vein; PA, pulmonary artery

**Fig. 2:**
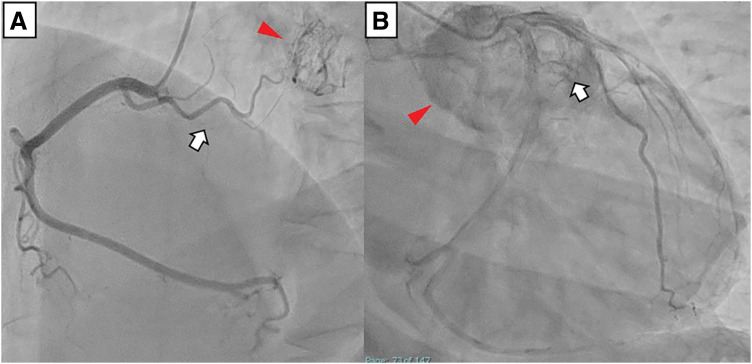
Preoperative coronary angiography demonstrating tumor-feeding vessels. (**A**) Right coronary angiography shows no significant stenosis and demonstrates a tumor-feeding branch arising from the right coronary artery (white arrow) with a corresponding tumor blush (red arrowhead). (**B**) Left coronary angiography shows no significant stenosis and demonstrates a tumor-feeding branch arising from the LAD (white arrow) with a prominent tumor blush (red arrowhead). LAD, left anterior descending artery

**Fig. 3:**
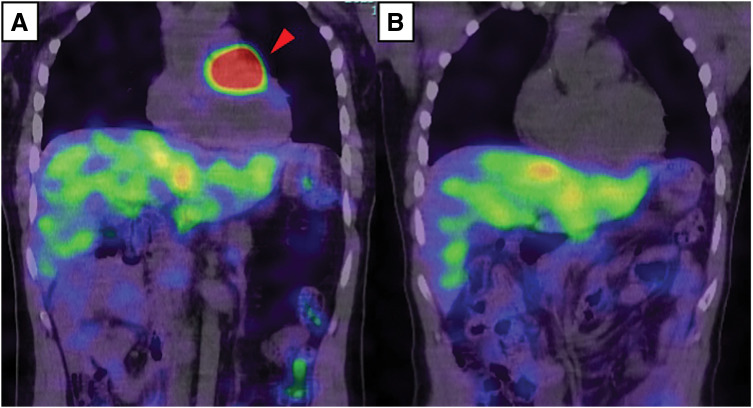
^123^I-MIBG SPECT/CT before and after surgery. (**A**) Preoperative fused coronal ^123^I-MIBG SPECT/CT demonstrates intense radiotracer uptake corresponding to the cardiac mass (arrowhead). (**B**) Postoperative fused coronal ^123^I-MIBG SPECT/CT shows complete resolution of abnormal uptake at the cardiac operative site. MIBG, metaiodobenzylguanidine; SPECT, single photon emission CT

On admission, he was hemodynamically stable with normal heart sounds and no signs of heart failure. Routine laboratory tests, including renal and liver function and brain natriuretic peptide, were unremarkable. Transthoracic echocardiography demonstrated normal left ventricular size and function without regional wall motion abnormalities or significant valvular disease; the left ventricular ejection fraction was approximately 60%. A lobulated heterogeneous mass measuring 4–5 cm was visualized between the ascending aorta and main pulmonary artery, extending toward the roof of the left atrium. Contrast-enhanced CT demonstrated a hypervascular mass on the left side of the aortic root between the ascending aorta and pulmonary artery (**Fig. 1A**), and 3D reconstruction clarified the relationship between the tumor and surrounding great vessels with prominent feeding arteries and draining veins (**Fig. 1B** and **1C**). Coronary angiography showed no significant coronary stenosis but revealed feeding arteries to the tumor arising from the LAD and RCA (**Fig. 2A** and **2B**). ^123^I-metaiodobenzylguanidine scintigraphy showed intense uptake corresponding to the cardiac mass (**Fig. 3A**) and increased uptake in the left adrenal gland, suggesting adrenal hyperplasia or a small pheochromocytoma. Preoperative catecholamine testing demonstrated a marked elevation of plasma noradrenaline (3.4 ng/mL), with low plasma adrenaline (0.02 ng/mL) and dopamine (≤0.02 ng/mL). Twenty-four-hour urinary testing showed a noradrenaline-dominant catecholamine excess (noradrenaline, 1580 μg/day; adrenaline, 11.3 μg/day; dopamine, 1100 μg/day), whereas other endocrine evaluations were unremarkable. Based on these findings, the patient was diagnosed with a functional cardiac paraganglioma adjacent to the LMT.

To prevent a perioperative catecholamine crisis, oral doxazosin was initiated and gradually titrated until adequate α-adrenergic blockade was achieved and blood pressure was stabilized. Elective surgery was then planned.

Under general anesthesia, median sternotomy was performed and an SVG was harvested from the right leg. After systemic heparinization, CPB was established with ascending aortic cannulation and venous drainage through the right atrium. Because preoperative imaging suggested close proximity to the LMT and radical excision could jeopardize left main coronary perfusion or necessitate coronary reconstruction, planned coronary revascularization was performed before tumor resection. Using an on-pump beating-heart approach, an SVG was anastomosed to the PL branch of the circumflex artery, and another SVG was anastomosed to the LAD. Given the potential risk of coronary compromise during oncologic dissection in this LMT-adjacent setting, we intentionally chose SVG conduits to the LAD and PL branch so that antegrade cardioplegia could be delivered selectively through the grafts if native coronary perfusion became unreliable. This ability to secure myocardial protection via the bypass grafts was a major reason we did not use the left internal thoracic artery in the present operation.

**Fig. 4:**
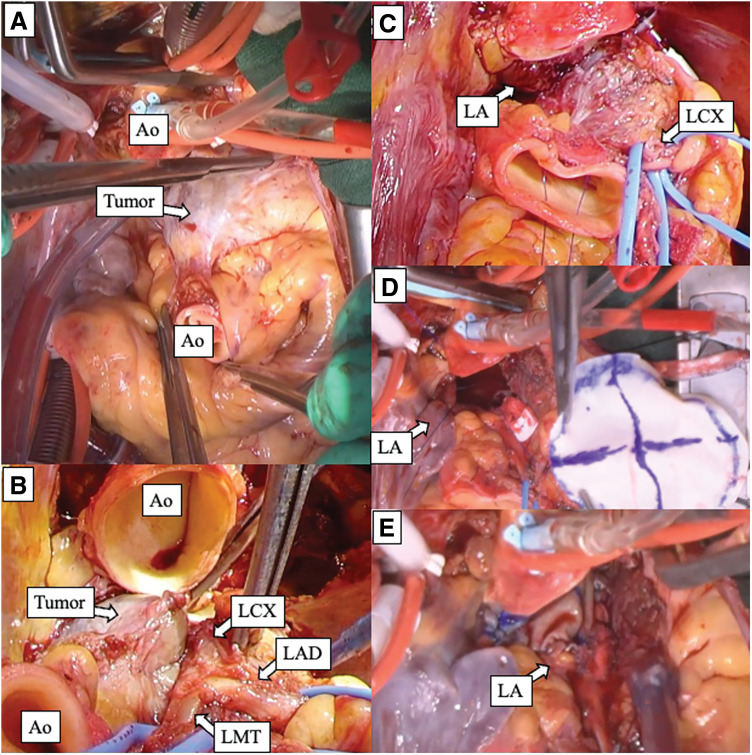
Intraoperative findings and stepwise surgical approach. (**A**) Surgical field after transection of the ascending Ao and main PA to obtain wide exposure of the tumor (arrow). (**B**) After dissection around the LMT, the LMT and LAD were successfully preserved, whereas the LCX was encased by the tumor. (**C**) Operative view after tumor excision showing the sacrificed LCX and the defect in the left atrial wall/roof created by en bloc resection. (**D**, **E**) Reconstruction of the left atrial defect using a bovine pericardial patch. Ao, aorta; LA, left atrium; LAD, left anterior descending artery; LCX, left circumflex artery; LMT, left main coronary trunk; PA, pulmonary artery

The ascending aorta was then cross-clamped, and antegrade cardioplegia was administered to achieve cardiac arrest. Because intraoperative findings suggested that the tumor involvement was confined to the LMT region, we did not perform selective graft cardioplegia. Instead, myocardial protection was maintained with intermittent retrograde cardioplegia administered approximately every 30 min, achieving adequate cardiac arrest and myocardial protection throughout the procedure. The tumor was located posterior to the main pulmonary artery and adjacent to the LMT. To obtain sufficient exposure, both the main pulmonary artery and ascending aorta were transected (**Fig. 4A**). The mass could be dissected away from the LMT and LAD, which were macroscopically intact; however, the left circumflex artery coursed through the tumor and could not be preserved (**Fig. 4B**). Therefore, the proximal and distal segments were clipped and divided. The tumor was firmly adherent to the roof of the left atrium; consequently, en bloc resection including a portion of the left atrial wall was required to achieve macroscopic complete resection (**Fig. 4C**). The resected specimen measured approximately 5 × 3 cm.

The defect in the left atrial roof was reconstructed with a bovine pericardial patch (**Fig. 4D** and **4E**). The main pulmonary artery and ascending aorta were reconstructed with end-to-end anastomoses. The proximal anastomosis of the SVG to the PL was created on the ascending aorta. After warm blood cardioplegia, the aortic cross-clamp was released and sinus rhythm resumed. Transit-time flow measurement confirmed satisfactory graft flow. Because the LAD had been preserved, the LAD graft was judged unnecessary and was ligated and divided near the distal anastomosis. CPB was discontinued.

Diffuse bleeding persisted from both the left atrial patch suture line and the extensive tumor dissection surface. In addition, the tumor appeared to have tumor-specific tissue fragility and focally extended into the left ventricular myocardium; therefore, the involved myocardium was resected with an adequate safety margin. As a result, troublesome bleeding from the myocardial cut surface further complicated hemostasis. Because the bleeding points were deep and the operative field was extremely limited, hemostatic maneuvers required repeated compression and retraction of the heart and adjacent coronary structures. Consequently, a second CPB run with brief repeat aortic cross-clamping was necessary to place additional sutures and secure hemostasis. After re-releasing the cross-clamp, the patient developed recurrent ventricular tachycardia and ventricular fibrillation requiring multiple defibrillations. We considered these arrhythmias to have been triggered by transient ischemia during hemostasis for bleeding from the myocardium in the LM–proximal LAD territory. In addition, because the myocardium had become markedly edematous after the prolonged cardiac arrest time, peripheral VA-ECMO was instituted via the right femoral artery and vein to stabilize hemodynamics and facilitate chest closure. With VA-ECMO support, hemostasis was achieved and the operation was completed.

Postoperatively, the patient was admitted to the ICU with VA-ECMO support. VA-ECMO was successfully weaned on POD 3. His postoperative course was complicated by ventilator-associated pneumonia due to methicillin-resistant *Staphylococcus aureus*, which was treated with broad-spectrum antibiotics and teicoplanin. He was extubated on POD 6, transferred to the general ward on POD 11, and discharged home on POD 28.

**Fig. 5:**
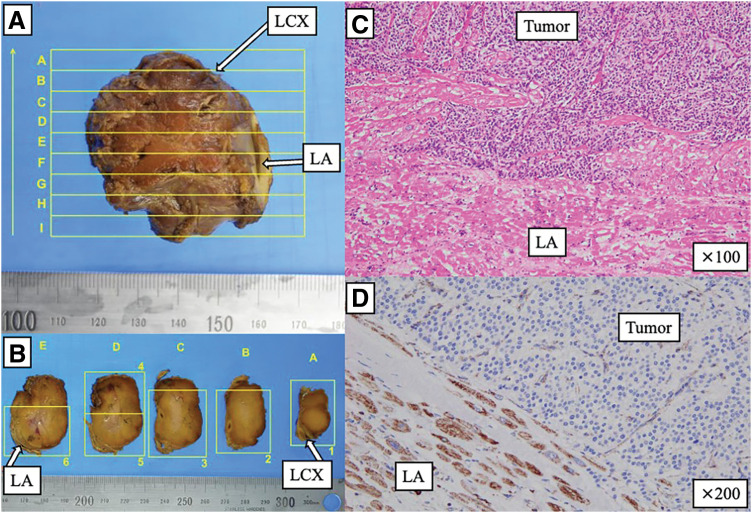
Gross and histopathological findings. (**A**) Gross specimen of the resected tumor; the regions adjacent to the LA and the LCX are indicated. (**B**) Serial sectioning of the specimen demonstrating the tumor cut surfaces and its relationship to the LA/LCX-adjacent regions. (**C**) Hematoxylin and eosin staining (×100) showing tumor infiltration into the left atrial wall (LA). (**D**) Immunohistochemistry for SDHB demonstrates loss of SDHB expression in tumor cells, with preserved staining in the adjacent left atrial myocardium serving as an internal positive control (×200). LA, left atrium; LCX, left circumflex coronary artery; SDHB, succinate dehydrogenase subunit B

Histopathological examination revealed a yellow-brown nodular tumor measuring 45 × 40 × 25 mm infiltrating the left atrial wall (**Fig. 5A**–**5C**). The tumor consisted of trabecular and nested proliferations of tumor cells with finely granular cytoplasm and intervening sinusoidal vessels. Although the tumor was in close proximity to the left circumflex coronary artery, there was no histopathological evidence of coronary arterial wall invasion. The surgical margins were negative for tumor (R0 resection). Immunohistochemically, SDHB immunostaining demonstrated loss of SDHB expression in tumor cells, with preserved staining in the adjacent left atrial myocardium, which served as an internal positive control (**Fig. 5D**). The Ki-67 labeling index was 12.2%. Loss of SDHB expression suggests an underlying alteration in SDHx (SDHA, SDHB, SDHC, SDHD, and SDHAF2), supporting the possibility of an SDHx-related susceptibility. According to the GAPP, the total score was 5, indicating a moderately differentiated tumor.

Postoperative urinary catecholamine and metanephrine levels normalized, and ^123^I-metaiodobenzylguanidine scintigraphy showed no abnormal uptake at the cardiac operative site (**Fig. 3B**). Persistent but unchanged uptake remained in the left adrenal gland without evidence of distant metastasis. Given the functional nature of the tumor, SDHB negativity, intermediate GAPP grade, family history of sudden death, and persistent adrenal uptake, close follow-up with regular endocrine testing and interval imaging was planned, and genetic counseling with next-generation sequencing for SDHx and related susceptibility genes was proposed. At the latest follow-up, the patient remained asymptomatic under outpatient surveillance.

## DISCUSSION

Primary cardiac tumors themselves are rare (reported prevalence 0.001%–0.03% in autopsy series), and cardiac paragangliomas constitute <1% of primary cardiac tumors. Contemporary series and reviews indicate that the vast majority are functional catecholamine-secreting lesions and are typically diagnosed in young to middle-aged adults (third to fifth decade).^1–3)^ Our patient’s paroxysmal hypertension, diaphoresis, and marked noradrenaline elevation were typical of catecholamine-producing disease. Because complete resection remains the only potentially curative option, operative planning must address 2 competing priorities: (1) oncologic radicality and (2) preservation of coronary perfusion under conditions of hypervascularity and limited surgical margins near the aortic root and proximal coronary arteries.^1–5)^

A central challenge in LMT-adjacent cardiac paraganglioma is determining whether a peel-off strategy can ensure true oncologic radicality while maintaining coronary perfusion. Even when imaging suggests a well-circumscribed mass, anatomically constrained regions may not provide a reliable dissection plane, and prioritizing preservation may leave residual disease because of microscopic invasion or a fibrous pseudocapsule.^5)^ In our case, the tumor was firmly adherent to the left atrial wall, and histopathology confirmed left atrial wall infiltration, supporting the oncologic rationale for planned en bloc excision with partial atrial wall resection. Thus, when an adequate margin cannot be confidently secured, a planned en bloc resection should be favored over insistence on tissue-preserving dissection.

The second pillar of the surgical strategy is myocardial protection—namely, maintenance of coronary perfusion during the highest-risk phase of dissection. In published cohorts, concomitant CABG or reconstruction of adjacent structures is required in a substantial proportion of patients, reflecting the frequent proximity of these tumors to the coronary arteries and great vessels.^1,3,5)^ Only a limited number of reports have described tumors involving or closely related to the LMT and its branches.^6–9)^ These reports suggest that coronary compromise may occur not only from direct involvement but also from manipulation, traction, or inadvertent injury during dissection in a restricted operative field. In our patient, preoperative imaging raised concern for close proximity to the LMT, and we anticipated that radical excision could jeopardize left main coronary perfusion. We therefore incorporated planned revascularization before tumor manipulation, constructing distal graft anastomoses on an on-pump beating-heart platform before cardioplegic arrest. We selected SVGs as the planned “safety bypass” because they also provided a reliable route for selective antegrade delivery of cardioplegia through the grafts if native coronary perfusion became compromised during tumor dissection. Although the internal thoracic artery is an excellent conduit in young patients, securing a rapid and dependable means of myocardial protection to both territories was prioritized in this operation. Establishing graft flow upfront provided a coronary “safety net” and converted a potential bailout into a proactive step, thereby expanding operative freedom during oncologic dissection. Although concomitant CABG has been reported in selected LMT-adjacent cases, the timing and stepwise sequence are often not explicitly detailed in the case literature.^6–9)^ Intraoperatively, the LMT and LAD were ultimately preserved and the LAD graft was ligated after confirming adequate native perfusion, illustrating how proactive revascularization planning can be individualized while prioritizing myocardial protection. Because the feasibility of preserving native left main coronary perfusion could not be reliably determined preoperatively, planned coronary revascularization was adopted as a proactive safety strategy rather than a bailout maneuver.

Adequate exposure was another decisive technical issue. The tumor was located posterior to the main pulmonary artery and adjacent to the LMT; therefore, direct visualization of the tumor–coronary–atrium interface was essential for safe and radical dissection. Cardiac autotransplantation with *ex situ* resection has been reported as an effective option for extensively invasive cardiac tumors because it provides excellent exposure;^10)^ however, it can be excessively invasive due to prolonged ischemic time and complex reconstruction. In our preoperative planning, autotransplantation was considered if the tumor extended to the posterior left atrium around the pulmonary venous confluence. In the present case, transection of the ascending aorta and main pulmonary artery under cardioplegic arrest provided sufficient exposure, allowing safe identification and preservation of the LMT and LAD and facilitating en bloc resection with partial left atrial wall resection and patch reconstruction. For selected LMT-adjacent lesions without extensive invasion of the posterior left atrium or pulmonary venous confluence, transection with primary end-to-end reconstruction of the ascending aorta and main pulmonary artery may therefore provide a less invasive alternative to autotransplantation while still enabling oncologically oriented radical resection.

This case also underscores the substantial perioperative bleeding risk associated with radical surgery for hypervascular functional cardiac paraganglioma. Preoperative α-adrenergic blockade is recommended for hormonally active pheochromocytoma/paraganglioma to reduce hypertensive crises and optimize volume status,^11–13)^ and our patient remained hemodynamically stable despite a prolonged operation. Nevertheless, diffuse massive bleeding from the extensive dissection surface and the left atrial patch suture line necessitated prompt reinstitution of CPB with brief repeat aortic cross-clamping to secure hemostasis. Importantly, contemporary surgical series still report non-negligible operative mortality, and fatal hemorrhagic complications have been documented,^1,3,4)^ highlighting the potentially lethal nature of hemorrhage in this setting. Accordingly, the key learning point is not ‘planning’ itself but the case-specific hemostatic pitfalls and techniques. In this setting, diffuse oozing from the broad hypervascular dissection surface and bleeding from the left atrial patch suture line may be compounded by tumor-specific tissue fragility and troublesome bleeding from the myocardial cut surface after partial left ventricular resection with an adequate safety margin. Practical measures include strict blood pressure control during hemostasis, reinforcement sutures with pledgets when appropriate for fragile atrial or myocardial edges, liberal use of topical hemostatic agents on broad raw surfaces, and a low threshold for prompt re-institution of CPB to secure a bloodless field and prevent progression of coagulopathy. In our case, recurrent malignant ventricular arrhythmias after weaning from CPB were considered to have been precipitated by transient ischemia during hemostasis for bleeding from the myocardium in the LM–proximal LAD territory. Moreover, because the myocardium became markedly edematous after the prolonged cardiac arrest time, rescue peripheral VA-ECMO was instituted to stabilize hemodynamics and facilitate chest closure. This support enabled completion of hemostasis and subsequent myocardial recovery, ultimately contributing to the favorable outcome.

Finally, pathological and genetic features are crucial for postoperative risk stratification. The tumor exhibited a Ki-67 labeling index of 12.2%, a moderately differentiated GAPP score (5 points), and loss of SDHB immunostaining, suggesting an SDHx-related susceptibility. Both higher GAPP grade and SDHB-deficient status have been associated with increased recurrence and metastatic potential even after apparently complete resection.^14–16)^ Although catecholamine levels normalized and no abnormal uptake was detected at the cardiac operative site on postoperative ^123^I-metaiodobenzylguanidine scintigraphy, persistent adrenal uptake and the family history warrant close endocrine surveillance, interval imaging, and genetic counseling with consideration of comprehensive susceptibility gene testing.

Taken together, this case illustrates a stepwise strategy to reconcile oncologic radicality with preservation of coronary perfusion in an LMT-adjacent functional cardiac paraganglioma. Planned revascularization before tumor manipulation provided a coronary “safety net” during the highest-risk phase, while transection of the ascending aorta and main pulmonary artery enabled sufficient exposure to preserve the LMT and LAD and accomplish en bloc resection with partial left atrial wall resection and patch reconstruction without resorting to cardiac autotransplantation. Nevertheless, as a single case, generalizability is limited, and the need for planned revascularization, graft targets, and the extent of exposure should be individualized according to tumor–coronary anatomy and the degree of invasion.

## CONCLUSIONS

LMT-adjacent functional cardiac paraganglioma poses a major surgical dilemma in which oncologic radicality must be balanced against preservation of coronary perfusion. Proactive CABG before tumor manipulation may provide a useful myocardial safety net and facilitate complete resection. In addition, case-specific hemostatic pitfalls—including fragile tissue, atrial reconstruction, and bleeding from myocardial resection surfaces—should be anticipated, and timely rescue mechanical circulatory support may be required when severe arrhythmia or myocardial edema compromises hemodynamic stability.

## DECLARATIONS

### Availability of data and materials

All data generated or analyzed during this study are included in this published article. Additional details are available from the corresponding author on reasonable request.
